# Lipocalin 2 induces neuroinflammation and blood-brain barrier dysfunction through liver-brain axis in murine model of nonalcoholic steatohepatitis

**DOI:** 10.1186/s12974-020-01876-4

**Published:** 2020-07-04

**Authors:** Ayan Mondal, Dipro Bose, Punnag Saha, Sutapa Sarkar, Ratanesh Seth, Diana Kimono, Muayad Albadrani, Mitzi Nagarkatti, Prakash Nagarkatti, Saurabh Chatterjee

**Affiliations:** 1 Environmental Health and Disease Laboratory, Department of Environmental Health Sciences, University of South Carolina, Columbia, SC 29208 USA; 2 Pathology, Microbiology and Immunology, University of South Carolina School of Medicine, Columbia, SC USA

**Keywords:** Lcn-2, MCD, NASH, NOX-2, Redox signaling, TLR4, NLRP3

## Abstract

**Background:**

Recent clinical and basic research implicated a strong correlation between NAFLD/NASH phenotypes with ectopic manifestations including neuroinflammation and neurodegeneration, but the mediators and critical pathways involved are not well understood. Lipocalin 2 (Lcn2) is one of the important mediators exclusively produced in the liver and circulation during NASH pathology.

**Methods:**

Using murine model of NASH, we studied the role of Lcn2 as a potent mediator of neuroinflammation and neurodegeneration in NASH pathology via the liver-brain axis.

**Results:**

Results showed that high circulatory Lcn2 activated 24p3R (Lipocalin2 receptor) in the brain and induced the release of high mobility group box 1 (HMGB1) preferably from brain cells. Released HMGB1 acted as a preferential ligand to toll-like receptor 4 (TLR4) and induced oxidative stress by activation of NOX-2 signaling involving activated p65 protein of the NF-κB complex. Further, the HMGB1-derived downstream signaling cascade activated NLRP3 inflammasome and release of proinflammatory cytokines IL-6 and IL-1β from brain cells. In addition, to advance our present understanding, in vitro studies were performed in primary brain endothelial cells where results showed high circulatory Lcn2 influenced HMGB1 secretion. Mechanistically, we also showed that elevated Lcn2 level in underlying NASH might be a likely cause for induction of blood-brain barrier dysfunction since the adipokine decreased the expression of tight junction protein Claudin 5 and caused subsequent elevation of pro-inflammatory cytokines IL-6 and IL-1β.

**Conclusion:**

In conclusion, the NASH-induced brain pathology might be because of increased Lcn2-induced release of HMGB1 and accompanying neuroinflammation.

## Background

Nonalcoholic fatty liver disease (NAFLD) or more recently reclassified as metabolic associated fatty liver disease (MAFLD) is a chronic liver disease that affects approximately 10–15% of US population and a significant number of the global population [1, 2]. According to the recent Centers for Disease Control and Prevention (CDC) reports, 90% of the obese population and 40–70% of the patients with T2DM in the USA are perceived to be suffering from MAFLD. MAFLD is characterized by excess fat accumulation in liver, and it may lead to nonalcoholic steatohepatitis (NASH) in many cases with scarring and inflammation in liver that consequently causes irreversible hepatic damage [3]. Previous studies have described that MAFLD/NASH contributes to disease pathology in several ectopic disorders like type 2 diabetes (T2DM), cardiovascular disease (CVD), and chronic kidney disease (CKD) [4]. In past few years, researchers also found evidence of neuroinflammatory disorders like Alzheimer’s disease (AD) with MAFLD/NASH phenotypes. MAFLD patients may have early or subtle cognitive dysfunction, including in the visuospatial and executive function domains [5]. A recent study by Seo et al. reported association of MAFLD with cognitive dysfunctions [6]. Also, Kim et al. showed that MAFLD accelerates the sign of Alzheimer’s disease (AD) in central nervous system (CNS) by inducing neuronal apoptosis and decreased expression of lipoprotein receptor 1 (LRP1) which is responsible for beta amyloid plaque clearance in AD [7]. Despite the existence of data showing the association of neuroinflammatory pathology in MAFLD or NASH, identifying factors and mechanisms of the underlying neuronal inflammation in MAFLD or NASH has remained elusive.

Previously, we and others showed that adipokines play a pivotal role in progression of MAFLD/NASH and subsequent ectopic manifestation underlying MAFLD/NASH [8–10]. Increased serum leptin has been correlated with hepatic inflammation and fibrosis in clinical studies [11]. Previously, we have showed that leptin induces oxidative stress and activates Kupffer cell that leads to hepatic fibrosis in NASH [12]. In a recent study, we discovered a mechanistic pathway mediated by adipokine leptin in renal inflammation in murine MAFLD model with progressive steatohepatitis [13]. Lipocalin2 (Lcn2) like leptin is another adipokine which is also associated with hepatic injury including acute and chronic inflammation, insulin resistance, and in lipogenesis [14]. Lcn2 is also known as neutrophil gelatinase-associated lipocalin (NGAL) which transports lipophilic molecules such as steroids, lipopolysaccharide, and iron in circulation and is considered as a prognostic biomarker of cirrhosis [15]. In a clinical study, Auguet et al. found that Lcn2 level is significantly higher in both liver and in circulation in female MAFLD patients compared to female with severe obesity and with non-significant liver disease [16]. Previous clinical studies have also implicated a strong correlation with ectopic diseases such as cardiovascular diseases, intestinal inflammation, neuroinflammation, and other metabolic disease such as T2DM and obesity with increased Lcn2 expression [17–20]. In CNS, Lcn2 has been reported to produce in low amount; indeed, it is quickly upregulated during neuroinflammation by different cell types in CNS (e.g., astrocytes, activated microglia, and endothelial cells) as these cells produce and possess receptors for Lcn2 [21–23]. Lcn2 contributes to immune responses that lead to pathogenesis of neurodegenerative diseases [24, 25]. However, the exact role of Lcn2 in disease prognosis is not clearly understood till date. Advancing the previous findings of ectopic manifestations of NAFLD and NASH, Eslam, M. and colleagues recently suggested metabolic associated fatty liver disease (MAFLD) as an appropriate acronym of NAFLD that describes more accurate symptoms associated with fatty liver disease [2].

The present study hypothesizes that increased hepatic and circulatory Lcn2 in NASH play an important role in neuroinflammation via the liver-brain axis. Using a murine model of NASH, we show that increased circulatory Lcn2 upregulates expression of Lcn2 receptor (24p3R) on brain cells and secretion of a damage associated molecular pattern protein (DAMP), a high mobility group box 1 (HMGB1) that subsequently induces oxidative stress and nod-like receptor protein 3 (NLRP3) inflammasome activation on brain cells. Furthermore, we show that increased circulatory Lcn2 causes blood-brain barrier (BBB) dysfunction by altered expression of tight junction protein Claudin5 and increases expression of proinflammatory cytokines in brain endothelial cells.

## Materials

### Animals

Pathogen-free, adult (8 weeks old), male C57BL/6 J wild-type (WT) mice (Jackson Laboratories, Ban Harbor, ME) were used in the study. The total number of animals in each group was assessed based on the calculations that ensured enough statistical power of 0.5. There were 6 mice per group that were allocated to their respective cages following the procedure of randomization. Mice were implemented in accordance with the NIH guideline for human care and use of laboratory animals and local IACUC standards. Procedures were approved by the University of South Carolina at Columbia, SC. Mice were fed with either CHOW or special diet at 22–24 °C with a 12-h light/12-h dark cycle with ad libitum access of food and water. All mice were sacrificed after animal experiments were done. Serum was prepared from blood and that has been freshly collected by cardiac puncture immediately after anesthesia, and kept at – 80 °C until analysis is required. Frontal cortex and liver from each mouse was collected after dissection, labeled properly, and fixed in Bouin’s (Sigma Aldrich, St. Louis, MO) solution or 10% neutral buffered formaldehyde respectively and further processed for immunostaining and visualizations. Tissues were also preserved for mRNA and protein extraction purpose.

### Diet-induced steatohepatitis mouse model (MAFLD/NASH)

Hepatic steatosis and inflammatory foci were induced in wild-type mice by feeding with methionine and choline deficient diet (MCD). In the absence of a definite model that fully represents human NASH, MCD diet mouse model was used as it is considered as one of the better models representing pathobiological mechanisms that cause human MAFLD to progress to advanced NASH [26].

### Isolation of blood-brain barrier endothelial cells from C57BL/6 J mice and treatment conditions

Endothelial BBB cells were isolated from adult (8–12 weeks old) C57BL/6 male mouse as described by Tobias Ruck [27]. Approximately, 10 mice were taken for the isolation purpose. Briefly, mice were sacrificed by cervical dislocation, and brains were kept in sterile PBS. Brain stems, cerebella, and thalami are being separated carefully by forceps, and meninges were removed by carefully rolling brain on sterile blotting paper. Brains were collected in 13.5 ml DMEM in 50 ml centrifuge tube. Brains were carefully minced with pipetting, digested with 0.6 ml collagenase CLS2 (10 mg/ml in DMEM) and 0.2 ml DNAse (1 mg/ml in PBS) in DMEM, and kept at orbital shaker at 37 °C and 180 rpm for 1 h. The tissue suspension was then mixed with 20% (w/v) BSA-DMEM, centrifuged at 1000×*g* 20 min at 4 °C to remove the myelin. Pellet was collected and resuspended 9 ml DMEM and add 1 ml collagenase/dispase (final concentration is 1 mg/ml) and 0.1 ml DNAse. The solution was digested in an orbital shaker for 1 h as described earlier. A Percoll gradient was prepared with mixing of 19 ml of sterile 1 × PBS, 1 ml of 10 × PBS, 1 ml of FBS, and 10 ml of Percoll, the mixture was filtered, and density gradient was settled by centrifuging at 3000×*g* for 1 h at 4 °C, with acceleration, without brake. Two milliliters of the digested mixture was carefully added to the top of the gradient solution and centrifuged at 700×*g* for 10 min at 4 °C without acceleration and brake. Approximately, 12 ml of the interphase was carefully collected and was transferred to a fresh 50-ml centrifuge tube, centrifuged at 1000×*g* for 10 min at 4 °C.

A plate was coated with fibronectin and collagen (1:1) ratio on the previous day of the isolation, washed with PBS, and isolated BBB endothelial cells were seeded in 60 mm tissue culture dish. The DMEM media was used for the growth purpose, supplemented with approx. 20% of FBS, basic fibroblast growth factor (bFgF) (20 μg/ml; approx. 0.05%), heparin (100 μl/ml; approx. 0.1%), and puromycin (4 mg/ml; approx. 0.1%). Puromycin was used only for the first 2 days of the culturing purposes. Cells were incubated at 37 °C in a CO2 incubator for growth purpose. Sterile new growth media was supplemented in every alternative day.

Cells were seeded onto 12 well tissue culture plates for experiment purpose, and growth was permitted to reach at 70% confluency before experiment. Cells have been kept for sera starvation (2% serum) for at least 18 h and treated with mouse recombinant Lcn2 (100 ng/ml), mouse recombinant leptin (100 ng/ml), and with the mixture of both leptin and Lcn2 (100 ng/ml each). All the preparation was made in 0.05% dimethyl sulfoxide (DMSO). The groups used for the experiment were control (0.05% DMSO), cells + mouse lipocalin2 (Lipcalin2), cells+ mouse leptin (leptin), and cells+ mouse lipocalin2+ mouse leptin (leptin+lipocalin2). The treatment was done for 12 h, supernatant was collected for enzyme-linked immunosorbent assay (ELISA), and cells were used for mRNA extraction. The in vitro experiments were performed 3 times.

In another set of experiment, cells were allowed to grow on confluence about 70% as described previously, exposed with mouse recombinant Lcn2 (100 ng/ml) or with 20 μl of serum from normal CHOW (CHOW) and MCD fed mouse or with DMSO (as vehicle only). Treatment was done for 24 h following 18 h sera starvation. Supernatant was collected for ELISA, and proteins were extracted by radioimmunoprecipitation assay (RIPA)-lysis buffer. Protein was measured by bicinchoninic acid assay (BCA) assay (Thermofisher Scientific, MA).

### siRNA inhibition of 24p3R (Lcn2 receptor) and treatment conditions

Actively growing primary mouse endothelial cells were seeded onto 12 well plates at low density for siRNA transfection. Mouse 24p3RsiRNA oligo duplex was purchased from Origene (Rockville, MD), and transfection was performed using 30 pmol of siRNA duplex in Viromer BLUE (BioNTech Protein, Germany) transection reagent following manufacturer’s protocol. Media containing the transfection reagents were replaced with fresh media after 4 h of transfection, mRNA was isolated on the following day, and 24p3R expression was studied for successful siRNA knock down. si-RNA-exposed primary brain endothelial cells were then treated with serum from CHOW or MCD fed mouse, or with recombinant Lcn2 as described previously. Protein was extracted, and supernatant was collected as described previously.

### Laboratory analysis

#### Immunohistochemistry

Paraffin-embedded tissues of frontal cortex or liver were prepared according to the standard protocol and sectioned to 5 μm thickness. The following sections were deparaffinized using standard laboratory protocol. Epitope retrieval of the deparaffinized sections was done using epitope retrieval solution and steamer (IHC-World, Woodstock, MD) for 45 min. Three percent of H_2_0_2_ was used for 15 min to block the endogenous peroxidases. After 10%, serum blocking primary antibody for mouse recombinant Lcn2 (R&D systems, Minneapolis, MN) and HMGB1 (Abcam, Cambridge, MA) was applied for both liver and brain slides in recommended dilutions and incubated overnight at 4°C. Only sections are labeled with phosphorylated-Tau (p-Tau), brain-derived neurotrophic factor (BDNF), and interleukin-6 (IL-6) and interleukin-1β (IL-1β) (Santa Cruz Biotechnology, Dallas, TX) were used overnight in recommended dilutions. Species-specific biotinylated conjugated secondary antibodies and streptavidin conjugated with horseradish peroxidase (HRP) were used to perform antigen-specific immunohistochemistry. 3,3’Diaminobenzidine (Sigma-Aldrich, St.Louis, MD) was used as a chromogenic substrate and counterstained with Mayer’s haematoxylin (Sigma-Aldrich). Sections were washed thrice with PBS and tween-20 (PBST-1X) between the steps. Sections were finally mounted in Aqua-Mount (Lerner Laboratories, Klamazoo, MI). Tissue sections were observed using an Olympus BX51 microscope (Olympus, USA) under a × 20 objective. CellSens Software from Olympus America (Centre Valley, PA) was used for morphometry analysis of images.

#### Immuno-fluorescence staining and microscopy

Formalin-fixed, paraffin-embedded brain tissue sections were subjected to deparaffinization using standard laboratory protocol. Epitope retrieval of the deparaffinized sections was done using epitope retrieval solution and steamer (IHC World) according to the manufacturer’s protocol. The primary antibodies such as anti-toll like receptor 4 (TLR4), anti-receptor for advanced glycation end products (RAGE), anti-HMGB1, anti-gp-91phox, anti-p47phox, anti-NLRP3, anti-ASC2, anti-Caspase1, anti- glial fibrillary acidic protein (GFAP), anti-cluster of differentiation molecule 11b (CD11b), and anti- platelet endothelial cell adhesion molecule-1 (PECAM1) (CD31) were used at recommended dilutions. Species-specific secondary antibodies conjugated with Alexa Fluor (633-red and 488-green) (Invitrogen) were used at recommended dilutions. The sections were mounted in a prolong diamond antifade reagent with 4’,6-diamidino-2-phenylindole (DAPI) (Life Technologies, Carlsbad, CA). Sections were observed under BX51 Olympus fluorescence microscope using × 20 and × 60 objective lenses. Morphometric analysis of the images was performed using the Cellsens software from Olympus America (Center Valley, PA).

### Western blot analysis

Protein samples from the tissues were extracted using RIPA-lysis buffer, quantified by BCA assay kit (Thermo Fisher Scientific, Rockford, IL). Approximately, 50 μg of lysate from CHOW and MCD groups were resolved by SDS PAGE, and protein bands were transferred to nitrocellulose membrane using pre-cut nitrocellulose/filter paper sandwiches (Bio-Rad Laboratories, Hercules, CA) and Trans-Blot Turbo transfer system (Bio-Rad). Primary antibodies were used at recommended dilutions, and species-specific HRP-conjugated secondary antibodies were used. Pierce-enhanced chemiluminescence (ECL) Western Blotting Substrate (Thermo Fisher Scientific, Rockford, IL) was applied for immunoreactivity detection. The blot was imaged using G: BoxChemi XX6 (Syngene imaging systems) and subjected to densitometry analysis using Image J. Primary antibodies HMGB1 (Abcam), mouse recombinant Lcn2 (R&D system), phosphor, and total p65 (Cell Signalling Technology) were used at 1:1000 dilutions. TLR4, RAGE, extracellular signal-regulated kinases 1/2 (ERK1/2), and p38, (Santa Cruz Biotechnology) were used at 1:500 dilutions, and β-actin was used at 1:10000. Compatible horseradish peroxidase-conjugated secondary antibodies (Abcam) at (1:5000) dilutions were used.

### Real-time quantitative polymerase chain reaction (PCR)

mRNA expression in the cerebral cortex and brain endothelial cells was examined by quantitative real-time PCR analysis. Total RNA was isolated from cerebral cortex and cells using TRIzol reagent (Invitrogen, Carlsbad, CA, USA) according to the manufacturer’s instructions and purified with the use of RNeasy mini kit columns (Qiagen, Valencia, CA, USA). cDNA was synthesized from purified RNA (1 μg) using iScript cDNA synthesis kit (Bio-rad, Hercules, CA, USA) following manufacturer’s instructions. Real-time quantitative polymerase chain reaction (qPCR) (qRTPCR) was performed with the gene-specific primers using SsoAdvanced SYBR Green Supermix and CFX96 thermal cycler (Bio-rad, Hercules, CA, USA). Threshold cycle (Ct) values for the selected genes were normalized against respective samples internal control 18S. Each reaction was carried out in triplicates for each gene and for each sample. The relative fold-change was calculated by the 2 − ∆∆Ct method. The sequences for the primers used for real-time PCR are provided in Table 1.

**Table 1:** Sequences of mouse primers used for real time-PCR

Gene name	Primer sequence (5′----3′)
IL-1r	Forward: GAATGACCCTGGCTTGTGTT Reverse: TGTGCTCTTCAGCCACATTC
TLR4	Forward: GGAGTGCCCCGCTTTCACCTC Reverse: ACCTTCCGGCTCTTGTGGAAGC
BDNF	Forward: TGCAGGGGCATAGACAAAAGG Reverse: CTTATGAATCGCCAGCCAATTCTC
RAGE	Forward: CCAATGGTTCCCTCCTCCTT Reverse: TAAGTGCCAGCTAAGGGTCC
TNFαR	Forward: GCTGTTGCCCCTGGTTATCT Reverse: ATGGAGTAGACTTCGGGCCT
p47phox	Forward: GGTCGACCATCCGCAACGCA Reverse: TGTGCCATCCGTGCTCAGCG
24p3R	Forward: TACCTGATGCGCCTGGAGCT Reverse: TTCTCCAGTTCCTGCAAAGCTT
Caspase1	Forward: TGGTCTTGTGACTTGGAGGA Reverse: TGGCTTCTTATTGGCACGAT
IL-18	Forward: AGCAGTGGTTTTCAGCTGGG Reverse: CACACCACAGGGGAGAAGTG
18S	Forward: TTCGAACGTCTGCCCTATCAA Reverse: ATGGTAGGCACGGCGACTA

### Enzyme-linked immunosorbent assay (ELISA)

Lcn2 and HMGB1 ELISA were performed with the sera collected from CHOW and MCD fed mouse groups. Mouse Lcn2 and HMGB1 ELISA kit were purchased from R&D Systems (Minneapolis, MN) and Abclonal Technology (Woburn, MA) respectively. IL-6 and IL-1β ELISA were performed with the collected supernatants from primary brain endothelial cells. IL-6 and IL-1β ELISA kits were purchased from ProteinTech (Rosemont, IL). ELISA was performed following manufacturer’s protocol.

### Statistical analysis

All in vivo and in vitro experiments were repeated 3 times. Statistical analysis was performed by unpaired, paired *t* test and analysis of variance (ANOVA) followed by Bonferroni post-hoc correction for intergroup comparisons. *P* < 0.05 value was considered as statistically significant.

## Results

### Upregulation of lipocalin 2 (Lcn2) in brain of NASH physiology

**Fig. 1:**
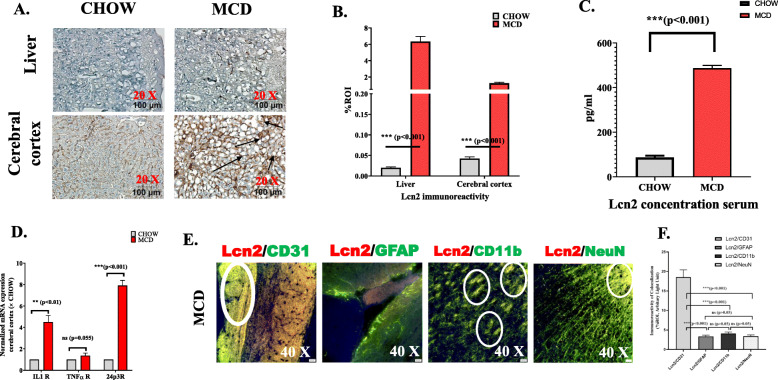
Lipocalin-2(Lcn2) upregulation nonalcoholic steatohepatitis (NASH) brain tissues. **a** Immunoreactivity (Lcn2) as shown by immunohistochemistry in liver and brain slices from mice fed with CHOW diet serves as a control and MCD diet (NASH). Images were taken at × 20 magnification (Scale 100 μm). **b** Morphometric analysis of Lcn2 immunoreactivity (mean data measured as arbitrary light units from three separate microscopic fields were plotted on y-axis) in CHOW and MCD fed mouse groups ( ***p < 0.001). C. Serum Lcn2 level in pg/mL was plotted as a bar graph with CHOW and MCD fed mice groups. **d** mRNA expression of receptors of IL-1, TNFα, and Lcn2 (24p3R) in CHOW and MCD fed mouse groups.**e**, **f** Co-localization and morphometric analysis of Lcn2 reactivity with astrocytes (GFAP), microglia (CD11b), brain endothelial cells (CD31)m and total neurons (NeuN)

Increased Lcn2 concentration in liver is reported in progressive MAFLD to NASH [15]. Our results implicated that there was significant increase in Lcn2 immunoreactivity in MCD fed mouse when compared to CHOW diet fed mouse (Fig. 1a). Notably, nearly 5-fold increased concentration of Lcn2 (487 pg/ml) was detected in the circulation of MCD mouse compared to CHOW in serum ELISA (Fig. 1c). We also studied mRNA expression of receptors for tumor necrosis factor α (TNFα), IL-1, and Lcn2 (24p3R) in cerebral cortex of CHOW and MCD fed mouse group. Results showed that interleukin 1 receptor (IL-1 r) (5-fold, *P* < 0.001, *n* = 3) and 24p3R (8-fold, *P* < 0.001, *n* = 3) (Fig. 1d) expressions were significantly increased in MCD fed mouse group compared to CHOW diet fed mouse group. However, no significant difference was observed for TNFα receptor’s mRNA expression. Immunohistochemistry with Lcn2 in frontal cortex showed significantly increased immunoreactivity in MCD fed mouse group compared to CHOW diet, representing an upregulation of Lcn2 in brain in MAFLD to NASH pathology (Fig. 1a, b). Furthermore, using immunofluorescence microscopy, we found that there was a significant increase co-localization of Lcn2 with both CD11b and CD31 especially at cerebral cortex and hippocampus regions respectively compared to GFAP positive cells in MCD diet mouse group (Fig. 1e) which confirms glial and endothelial location of Lcn2 in NASH pathology.

### Increased Lcn2 in brain of MAFLD and progressive NASH is correlated with neuroinflammation and neurodegeneration

**Fig. 2:**
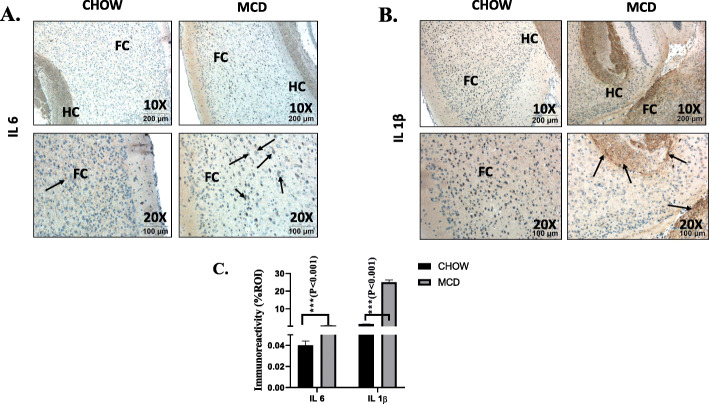
Upregulated Lcn2 in brain is correlated with neuroinflammation A and B. Immunoreactivity of neuroinflammatory markers IL-6 (**a**) and IL-1β (**b**) proteins were shown by immunohistochemistry in brain slices (focused area is cerebral cortex) from mice fed with CHOW diet serves as a control and MCD diet (NASH). Immunoreactivity was indicated by black arrows. Images were taken at × 10 and × 20. Frontal cortex is marked as FC, and HC is hippocampus. **c**. Morphometric analysis of IL-6 and IL-1β was carried out as mean data from three separate microscopic fields (plotted on y-axis ) of CHOW and MCD fed mouse groups. Data were expressed as %ROI (mean ± SD, *n* = 3)

IL-6 and IL-1β expression was examined in brain tissues to study neuroinflammation in MAFLD/NASH pathology. Microglial activation in brain leading to M1 polarization and production of proinflammatory cytokines like IL-6, IL-1β, and TNFα has been reported previously [28]. In the present study, we also observed an increased IL-1r expression that prompted us to check expressions of IL-1β in the brain tissues of mice that had NASH. Results showed marked increases in mRNA expression of both IL-6 (5-fold, *p* < 0.001, *n* = 3) and IL-1β (12-fold, *p* < 0.001, *n* = 3) in MCD (NASH) fed mouse group as compared to CHOW diet mouse group (Fig. 2b). Notably, immunohistochemistry results showed a 10-fold increased IL-6 expression (*p* < 0.01, *n* = 3) and more than 20-fold increased IL-1β expression in MCD fed mouse group as compared to CHOW diet group (Fig. 2b). These results clearly depicted neuroinflammation in MAFLD pathology albeit in a mouse model.

**Fig. 3:**
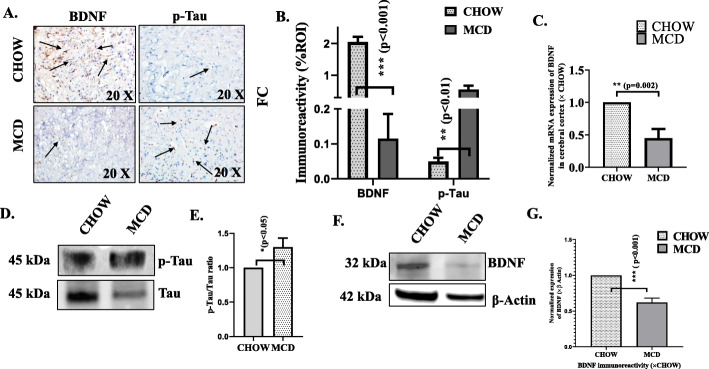
Upregulated expression of Lcn2 is correlated with neurodegeneration. **a** Neurodegenerative markers BDNF and phosphorylated Tau protein (Serine 396) were shown by immunohistochemistry in brain slices (focused area is cerebral cortex) from mice fed with CHOW diet serve as a control and MCD diet (NASH). Immunoreactivity was indicated by black arrows. Images were taken × 20 magnification. **b** Morphometric analysis of BDNF and pTau immunoreactivity (mean data measured as arbitrary light units from three separate microscopic fields were plotted on y-axis) in CHOW and MCD fed mouse groups (***p < 0.001). **c** mRNA expression analysis of BDNF in cerebral cortex of CHOW and MCD fed mouse groups. mRNA expression was assessed by quantitative real-time PCR, and expression was normalized against CHOW diet group, *p < 0.05, **p < 0.01, ***p < 0.001. **d** Immunoblot analysis with pTau and Tau proteins from cerebral cortex from CHOW and MCD fed mouse groups. **e** Densitometric analysis of pTau reactivity calculated as pTau and Tau ratio, displayed as mean ± SD (*n* = 3) and plotted as bar graph, and statistical significance was tested. **f** Immunoblot analyses of BDNF from cerebral cortex of CHOW and MCD mouse groups. **g** Densitometric analyses of BDNF immunoreactivity displayed as mean ± SD (*n* = 3), normalized against β-actin and plotted as bar graph

To study neurodegeneration in MCD fed mice, BDNF expression and phosphorylation of Tau proteins were studied. BDNF is principal marker for neuroplasticity that regulate structural and functional effects on excitatory and inhibitory synapses Decreased expression of BDNF is a well-established finding during neurodegeneration [28]. On the other hand, Tau is a microtubule associated protein that stabilizes neuronal microtubules and promotes axonal growth [29]. Tau aggregation is characteristic of several neurodegenerative diseases including AD [30]. Hyper phosphorylation of Tau protein is the hallmark for AD and AD like symptoms. Our results showed that there was significantly decreased BDNF gene expression (to nearly 50%, *p* < 0.01, *n* = 3) (Fig. 3c) and protein expression, as indicated by immunoreactivity in immunohistochemistry (to nearly 2.5%, *p* < 0.001, *n* = 3) (Fig. 3a, b) and immunoblot analysis (to nearly 50%, *p* < 0.001, *n* = 3) ( Fig. 3f, g) in brain tissue of MCD diet mouse group (NASH) compared to CHOW fed mice (lean control). Remarkably, more than 15-fold increased immunoreactivity of phosphorylated Tau protein (phosphorylated at serine 396) was observed in brain tissue of MCD diet mouse group compared to CHOW diet fed (Fig. 3a, b) mouse group suggesting a clear implication of neurodegeneration and signs of AD in NASH murine model in the presence of increased circulatory Lcn2.

### Increased Lcn2 is correlated with increased secretion of HMGB1 from brain cells

**Fig. 4:**
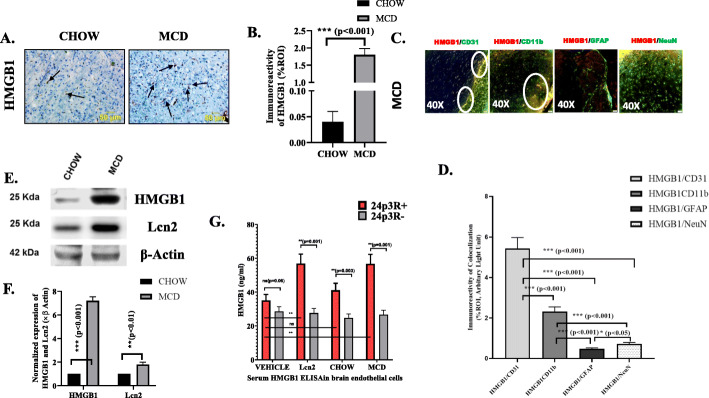
Lcn2 is correlated with increased HMGB1 secretion from brain cells. **a** Immunoreactivity of HMGB1-DAMP as shown by immunohistochemistry in brain slices from mice fed with CHOW diet serves as a control and MCD diet as NASH. Images were taken in × 20 in cerebral cortex area of brain. Immunoreactivity was indicated with black arrows. **b** Morphometric analysis of HMGB1 immunoreactivity in CHOW and MCD fed mouse groups. Morphometric analysis was performed by taking mean % ROI values from three separate fields (designed on y-axis). **c** Co-localization and morphometric analysis of HMGB1 reactivity with endothelial cells, microglia, astrocytes, and neurons marked as HMGB1/CD31, HMGB1/CD11b, and HMGB1/GFAP. HMGB1/NeuN in brain slices from MCD mouse group. Co-localization was displayed yellow dots in C, highlighted by white circles. Images were taken at × 40. Significance was tested between the groups by unpaired *t* test, *p < 0.05, **p < 0.01, ***p < 0.001. **e** Immunoblot analysis of HMGB1 and Lcn2 with protein extracts from cerebral cortex from CHOW and MCD fed mouse group. **f** Morphometry analysis of HMGB1 and Lcn2 immunoblot with protein extracts from cerebral cortex, normalized against β-actin, and plotted as bar graph. **g** HMGB1 ELISA was performed with supernatants from brain endothelial cells (24p3R+), and 24p3RsiRNA exposed brain endothelial cells (24p3R−) treated with VEHICLE (0.05% DMSO), mouse recombinant Lcn2 (100 ng/ml), 20 μl of serum from CHOW, and MCD fed mouse. HMGB1 concentration (ng/ml) was plotted as bar graph. Significance was tested by unpaired *t* test and pair *t* test between the groups of 24p3R+ and 24p3R− ,*p < 0.05, **p < 0.01, ***p < 0.001, ns, nonsignificant; *p* ≥ 0.05

Following our observation of a positive correlation with increased Lcn2 and neuroinflammation in NASH mouse, we were further interested to study expression of HMGB1 in brain tissues of NASH mouse group. HMGB1 is a DAMP released from nucleus upon tissue injury and inflammation [31, 32]. Previously, we showed that HMGB1 is released from fatty liver and induced oxidative stress and inflammation [33]. Results in the present study showed a significant increase in HMGB1 concentration in the cerebral cortex of MCD fed mouse group. Immunohistochemistry of brain tissues for HMGB1 showed at least 25-fold increased immunoreactivity of the said protein in cerebral cortex region (Fig. 4a, b). Immunoblot of protein extracts from cerebral cortex of both CHOW and MCD diet fed mouse group showed a 7-fold increased expression of HMGB1 (***p < 0.001, *n* = 3) and a 2-fold increased expression of Lcn2 protein (**p < 0.01, *n* = 3) (Fig. 4,e f). Furthermore, immunofluorescence studies of HMGB1 with brain cell specific markers CD11b, GFAP, CD31, and NeuN showed significantly increased co-localization of HMGB1 with CD31 and CD11b confirmed glial and endothelial localization of HMGB1 similar to Lcn2 in NASH (Fig. 4c, d). All the above results indicated a positive correlation of increased Lcn2 and HMGB1 secretion in the brain of NASH mouse.

To study whether Lcn2 induced the secretion of HMGB1, experiments were performed in mouse primary brain endothelial cells isolated from adult male C57BL/6c mouse and primed with recombinant Lcn2 (100 ng/ml). To study the effect of circulatory Lcn2 from mouse NASH models, 20 μl of serum from both CHOW and MCD diet mouse was treated to the isolated endothelial cells to mimic the ex vivo condition. HMGB1 concentration was detected by ELISA. Results showed that there was a significantly increased HMGB1 secretion following incubation with either recombinant Lcn2 or with serum from MCD fed mouse group when compared to vehicle control. Notably, a significant difference of HMGB1 secretion was not detected in serum from CHOW diet fed mouse group when compared with the vehicle control (Fig. 4g). Role of Lcn2 in HMGB1 induction was further investigated by exposing primary brain endothelial cells with 24p3RsiRNA with or without recombinant Lcn2 and with sera from both CHOW and MCD fed mouse group. Results showed that HMGB1 secretion was significantly decreased in siRNA exposed cells, treated with serum from CHOW, MCD fed mouse, and with recombinant Lcn2 compared to wild-type cells (Fig. 4g). The data suggested that Lcn2 played a crucial role in inducing HMGB1 secretion from brain in NASH phenotypes.

### HMGB1 release is associated with TLR4, RAGE, and NADPH oxidase 2 (NOX-2) signaling pathway to induce oxidative stress and inflammation in the brains of NASH-mice

**Fig. 5:**
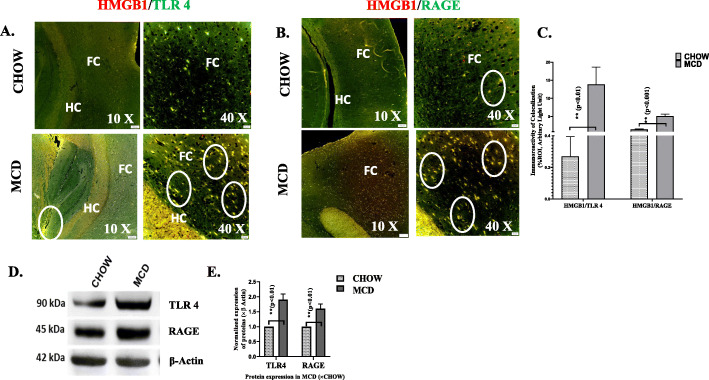
HMGB1 activates TLR4 and RAGE signaling pathway to induce oxidative stress and inflammation in brain of MAFLD. **a** Co-localization of HMGB1/TLR4 and in **b** HMGB1/RAGE of CHOW and MCD mouse groups. Images were taken at × 10 and × 40 magnification. Co-localization of HMGB1 and TLR 4 was displayed as yellow dots, highlighted by white circles. **c** Immunoreactivity was displayed as bar graph representing mean ± SD of ROI % of 3 different areas (*n* = 3). Significance was tested by unpaired *t* test,*p < 0.05, **p < 0.01, ***p < 0.001. -.**d** Immunoblot analysis of TLR and RAGE from cerebral cortex of CHOW and MCD fed mouse groups. **e** Morphometric analysis of immunoblots **a**–**c** displayed as mean ± SD (*n* = 3), normalized against β-actin, and plotted as bar graph. Significance was tested by unpaired *t* test, *p < 0.05, **p < 0.01, ***p < 0.001

**Fig. 6:**
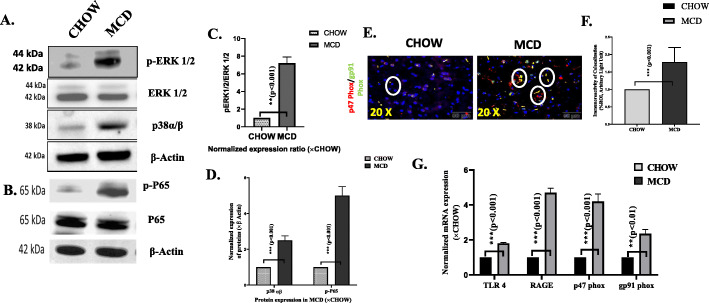
Immunoblot analysis of proteins extracted from cerebral cortex from CHOW and MCD mouse groups. **a** Proteins resolved MAPK activation markers phospho and total ERK1/2 and p38α/β and NF-κβ activation marker phospho-p65 and total p65 in **b**. **c** Densitometric analysis of phospho ERK½ and total ERK½ is displayed as mean ± SD (*n* = 3). **d** Densitometric analysis of p38α/β and NF-κβ activation marker phospho-p65 and total p65 normalized against β-actin and plotted as bar graph. **e** Co-localization and morphometric analysis p47Phox/gp91Phox as shown by immunofluorescence imaging in brain sections from CHOW and MCD fed mouse groups. Co-localization was displayed yellow dots, indicated by white circles. Images were taken at × 20 magnification. **f** immunoreactivity was displayed as bar graph representing mean ± SD of ROI % of 3 different areas (*n* = 3). Significance was tested by unpaired *t* test,*p < 0.05, **p < 0.01, ***p < 0.001. **g** mRNA expression analysis of TLR4, RAGE, and p47 Phox in cerebral cortex of CHOW and MCD fed mouse groups. mRNA expression was assessed by quantitative real-time PCR, and expression was normalized against CHOW diet group, *p < 0.05, **p < 0.01, ***p < 0.001

Previous evidence suggests that HMGB1 is primarily responsible for pathogenesis in chronic and acute liver injury [34, 35]. Actively and/or passively secreted HMGB1 signals through RAGE and toll-like receptors such as TLR2 and TLR4 to induce immunological responses and cytokine release [36–38]. We studied the expression levels of TLR4 and RAGE receptors in protein and mRNA levels. mRNA and protein expressions results showed significantly increased expression of both TLR4 and RAGE receptors in MCD mouse group when compared to CHOW diet group (Fig. 5d, e, g). Association of HMGB1 with TLR4 or RAGE receptors was confirmed by co-localization analysis using the Olympus software following fluorescence microscopy. Dual fluorescent labeling was used to stain HMGB1 (red) and RAGE or TLR4 (green) to confirm their co-localizations (yellow) with HMGB1 (Fig. 5a, b). Results showed that both HMGB1-RAGE (*p* < 0.001, *n* = 3) and HMGB1-TLR4 (*p* < 0.01, *n* = 3) interactions were significantly high in the cerebral cortex and hippocampus of NASH mouse brain (Fig. 5b). Previous studies reported that HMGB1 acted as potent inducers of oxidative stress in liver during MAFLD [39]. MCD fed mouse group representing NASH showed at least a 2-fold increased co-localization events of p47phox and gp91 phox when compared to lean control (CHOW diet) (*p* = 0.007) (Fig. 6e, f) suggesting a strong NADPH oxidase 2 (NOX-2) activation. mRNA expressions of p47phox gene in cerebral cortex were significantly higher in MCD fed mouse group when compared to CHOW diet mouse group, thus confirming NOX-2-mediated oxidative stress in the brain tissues of MAFLD mice (Fig. 6g). Oxidative stress has been reported to activate of inflammatory signaling cascades such as MAP kinase and NF-κB [40, 41]. To show that increased release of HMGB1, increased activation TLR4/RAGE also correlated with downstream oxidative stress signaling, and expressions of p38 MAPK and ERK components were examined. Results showed that there was a subsequent increase in expression of p38 MAPK (2.5-fold, *p* < 0.001), *n* = 3), ERK 1/2 (7-fold, *p* < 0.001, *n* = 3), and p65 phopsphorylation (4-fold < 0.001, *n* = 3) in MCD fed mice when compared to CHOW diet mice (Fig.6 a, d). Taken together, the above results demonstrated that higher release of HMGB1 correlated strongly with neuroinflammatory signaling cascade possibly via targeting TLR4 and RAGE receptors in cerebral cortex of murine MAFLD model.

### Activation of NLRP3 inflammasome in brain tissues of murine NASH model

**Fig. 7:**
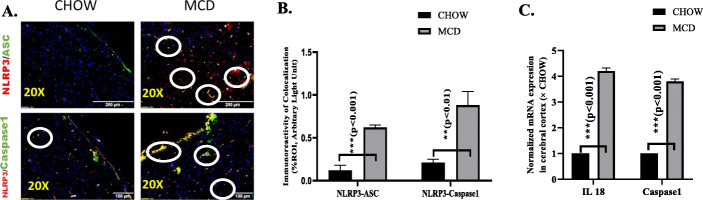
Activation of NLRP3 inflammasome in brain tissues of murine MAFLD model. **a**, **b** Co-localization and morphometric analysis of NLRP3/ASC and NLRP3/Caspase1 as shown by immunofluorescence imaging in brain sections from CHOW and MCD fed mouse groups. Co-localization was displayed yellow dots, indicated by white circles. Images were taken at × 20. Immunoreactivity of co-localization was displayed by yellow dots in **a**, and immunoreactivity was displayed as bar graph representing mean ± SD of ROI % of 3 different areas (*n* = 3). **c** mRNA expression was assessed by quantitative real-time PCR, and expression was normalized against CHOW diet group, *p < 0.05, **p < 0.01, ***p < 0.001

Oxidative stress and NF-κB activation have been shown to drive NLRP3 inflammasome activation to induce inflammation [42]. Active NLRP3 inflammasome binds with its adaptor protein ASC that comprises of caspase and pyrin recruitment domains. Caspase1 protein subsequently cleaves pro-IL-1β and Pro-IL-18 to form active IL-1β and IL-18 [43, 44]. NLRP3 inflammasome activation was studied by immunofluorescence assay. Interaction of NLRP3 with caspase 1 or ASC2 was confirmed by co-localization studies (yellow dots) (Fig. 7a). Results showed that there was at least a 4-fold increased co-localization of both NLRP3-caspase1 and NLRP3-ASC2 detected in the cerebral cortex of murine MAFLD model when compared to lean control mice (Fig. 7b). Abundance of caspase1, IL-1β, and IL-18 has been identified for neuroinflammatory and neurodegenerative diseases and is associated with neuropathology. We studied mRNA expression of caspase1, IL-1β, and IL-18, and results showed an increased expression of the above genes in cerebral cortex of MCD fed mouse group when compared to the CHOW diet mouse group (Fig. 7c). The above results implicated that activation of NLRP3 inflammasome is responsible in part for neuroinflammation and neurodegeneration in NASH murine model.

**Fig. 8:**
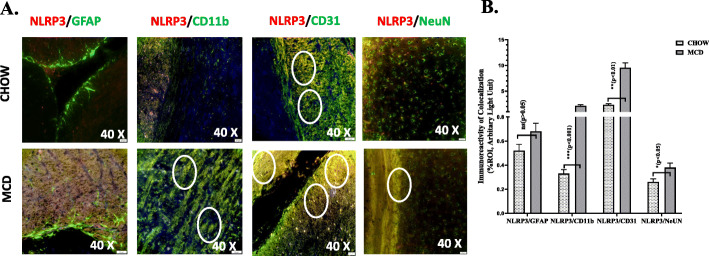
**a** Co-localization and morphometric analysis of NLRP3 reactivity with astrocytes, microglia, neurons, and brain endothelial cells marked as NLRP3/GFAP, NLRP3/CD11b, NLRP3/NeuN, and NLRP3/CD31 respectively in brain slices from MCD fed mouse group. Co-localization was displayed yellow dots in **a**, highlighted by white circles. Images were taken at × 40. **b** Immunoreactivity of co-localization was displayed by yellow dots in **a**, and immunoreactivity was displayed as bar graph representing mean ± SD of ROI % of 3 different areas (*n* = 3). Significance was tested between the groups by unpaired *t* test, *p < 0.05, **p < 0.01, ***p < 0.001

Further, we were curious to study localization of NLRP3 inflammasome complex in brain cell types such as astrocytes, microglia, neurons, and brain endothelial cells of both CHOW and MCD mouse groups. Immunofluorescence was performed by using dual label staining with NLRP3 (red) and GFAP or CD11b or CD31or NeuN (green) to stain astrocytes, microglia, brain endothelial cells, and total neurons respectively (Fig. 8a). Results showed that there was 6-fold and 7-fold increased co-localization of NLRP3 with CD11b and CD31 respectively in NASH mouse model compared to lean control (Fig. 8b). These results confirmed a formation of active inflammasome complex in blood-brain barrier and in microglial cell types in the brain of murine MAFLD model.

### High circulatory Lcn2 caused blood-brain barrier dysfunction

**Fig. 9:**
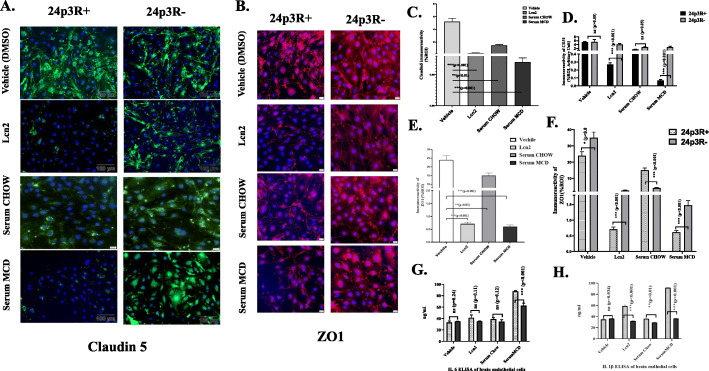
High circulatory Lcn2 causes blood brain barrier dysfunction. **a**, **b** Immunoreactivity of Claudin 5 (green) in **a** and Zonula Occludens (ZO1) (Red) in **b** as shown by immunofluorescence microscopy in mouse primary brain endothelial cells exposed with or without siRNA-24p3R and treated with VEHICLE (DMSO), Mouse recombinant Lcn2 (100 ng/ml), 20 μl of serum from CHOW, and MCD fed mouse. Nucleus was stained with DAPI. Images were taken at × 20 magnification. **c**, **e** Morphometry analysis of Claudin 5 (**c**) and ZO1 (**e**) immunoreactivity in brain endothelial cells without exposure of 24p3R siRNA. Immunoreactivity was displayed as bar graph representing mean ± SD of ROI % of 3 different areas (*n* = 3). **d**, **f** Morphometric analysis of Claudin 5 immunoreactivity in **d** and ZO1 in **f** in primary brain endothelial cells exposed with or without 24p3RsiRNA. Data was calculated as mean ± SD of %ROI of 3 different fields and displayed as bar graph. **g**, **h** IL-6 and Il -1β levels in pg/ml in supernatants from brain endothelial cells were displayed by bar graph. Data was calculated as expressed as mean ± SD, and significance was calculated by paired *t* test between the groups, *p < 0.05, **p < 0.01, ***p < 0.001. All statistical analysis was performed by unpaired and paired *t* test between the means. Significance was tested by paired test between the groups. *p < 0.05, **p < 0.01, ***p < 0.001, followed by Bonferroni post-hoc corrections

We already showed in the previous datasets that high circulatory Lcn2 in MAFLD induces HMGB1 secretion in primary brain endothelial cells. To show that increased Lcn2 caused blood-brain barrier integrity loss, primary brain endothelial cells were primed with mouse recombinant Lcn2 (100 ng/ml) and/or with sera from CHOW and MCD fed mouse group as described previously. si RNA knock down of Lcn2 receptor (24p3R) was performed by exposing cells with 24p3RsiRNA duplex (24p3R−) as described in methods. BBB dysfunction was studied by checking the expression of tight junction proteins Claudin 5 and Zonula Occludens 1 (ZO1) and performing ELISA with IL-6, IL-1β, and TNFα from the collected cell supernatants, treated for 24 h. Immunofluorescence results showed that Claudin 5 expression was significantly decreased in cells that were exposed with mouse recombinant Lcn2 (100 ng/ml) (to nearly 6.7%, *p* < 0.001, *n* = 3) and serum from MCD mouse (to nearly 2.5%, *p* < 0.001, *n* = 3). ZO1 expression was also decreased significantly with recombinant Lcn2 and with serum from MCD group (Fig. 9b, e). Most interestingly, serum from the MCD mouse group containing high circulatory Lcn2 showed more potency in terms of Claudin 5 inhibition compared to mouse recombinant Lcn2 (3-fold, *p* < 0.001, *n* = 3) (Fig. 9a, c). Role of circulatory Lcn2 in Claudin 5 and ZO1 expression was studied by exposing brain endothelial cells with 24p3Rsi RNA duplex and with serum from CHOW or MCD or with mouse recombinant Lcn2. All data were compared between 24p3R+ and 24p3R− groups. Results showed an 8-fold and 30-fold increased expression of Claudin 5 and 4-fold and 3-fold increased expression of ZO1 with mouse recombinant Lcn2 and serum from MCD respectively in 24p3R- cells as compared to 24p3R+ cells (*p* < 0.001, *n* = 3) (Fig. 9d, f). The above results were further confirmed by performing an immunoblot for Claudin 5 (Supplementray Figure 2). Proinflammatory cytokine secretion IL-6 and IL-1β was quantified by ELISA in the cell culture supernatants to ascertain the role of Lcn2. Results showed that there was an increased secretion of both the cytokines IL-6 and IL-1β following incubation with serum from MCD mouse group while it was significantly decreased in 24p3RsiRNA exposed cells. In conclusion, the above results represented that high circulatory Lcn2 in MAFLD/NASH induces blood-brain barrier disruption by altering expression of tight junction proteins Claudin 5, ZO1, and induced the secretion of proinflammatory cytokines IL-6 and IL-1β in brain endothelial cells.

## Discussion

MAFLD or its progressive inflammatory counterpart NASH has been shown to have ectopic manifestations in different organ systems. In the current study, using a murine model of progressive MAFLD pathology that represents closely nonalcoholic steatohepatitis (NASH), we show that there was a marked increase of Lcn2 in systemic circulation and in diseased livers. The higher Lcn2 caused a strong induction of HMGB1 in the liver and in the frontal cortex and was concomitantly associated with proinflammatory surge in mediators in brain pathology, subsequent decrease in neurotropic factor BDNF, and an increase in Tau protein. As an adipokine, Lcn2 plays a role in diverse biological functions in the CNS [45]. Though precise role of Lcn2 in pathophysiology of CNS remains to be outlined, studies have found some correlation with increased Lcn2 and CNS disorders. Lee et al. suggested that Lcn2 may act as a pro-apoptotic factor by sensitizing microglia to apoptotic stimuli and that facilitates morphological transformation and self-regulatory apoptotic elimination [21]. Lcn2 is also thought to induce astrocytic neurotoxicity and neurosecretion by several research groups [46, 47]. Although Lcn2 secretion and its concentrations are low in normal physiological conditions, brain injury or infection could promote Lcn2 secretion and increased expression of cationic Lcn2 receptor (24p3R) in astrocytes, activated microglia, and endothelial cells in CNS [22, 23]. The present study shows high circulatory Lcn2 in murine NASH, and these levels in the circulation may induce expression of inflammatory receptors and Lcn2 receptor 24p3R in brain tissues of NASH mice, thus raising the possibility of an altered neuroinflammatory pathology in the brain of NASH mice.

Previous studies reported that excessive inflammatory responses are detrimental for neuronal regeneration and that impair the neuronal circuit in the CNS [48, 49]. Chronic neuroinflammation is critical for the onset and the progression of neurodegenerative diseases including AD [50]. Our current research shows increased secretion of proinflammatory cytokines like IL-6 and IL-1β with observable clinical signs of neurodegeneration under NASH pathological condition. Also, expressions of BDNF and p-Tau protein, crucial mediators of neuronal plasticity/neurogenesis, and neurodegeneration respectively were critically altered in NASH mice. Results showed decreased BDNF mRNA and protein expression and increased aggregation of p-Tau in cerebral cortex of murine NASH. BDNF maintains neuronal plasticity, survival, and neurogenesis that is known to play an important role in survival of neurons in both peripheral and central nervous system [51]. Decreased BDNF mRNA and protein expression has been exclusively reported in multiple brain locations of AD patients [52]. Aggregation of p-Tau is also considered as a hallmark for AD pathological symptoms [53, 54]. Based on the above evidence, the current results in our study clearly implicated a positive correlation of NASH with AD like symptoms in murine NASH model. Our study corroborates a previous study by Kim et al. where they also reported signs of neurodegeneration and appearance of AD like symptoms in NASH mouse model but the relationship with Lcn2 as a possible mediator was not shown [7].

To establish a possible mechanism for an increased Lcn2 mediated brain pathology in NASH, we studied the expression of HMGB1, a DAMP in brain tissue of both CHOW and MCD fed mouse group to ensure whether increased Lcn2 influences HMGB1 induction and activation of subsequent inflammatory signaling pathways. HMGB1 is released from numerous cells types throughout the body, including cells from nervous system following tissue injury [55, 56]. Previously, He at al. showed that HMGB1 may causes blood-brain barrier disruption leading to cognitive deficits in aged rats [57]. Our in vivo results implicated positive correlation of increased Lcn2 and HMGB1 secretion in brain of NASH murine model. Moreover, our results also showed that circulatory Lcn2 influenced HMGB1 release from blood-brain barrier endothelial cells, an observation that might suggest altered pathology and poor outcome of barrier integrity. Interestingly, serum from MCD mouse group that showed NASH pathophysiology and containing high circulatory Lcn2-induced HMGB1 secretion in brain endothelial cells in 24 h while it was significantly decreased in 24p3RsiRNA-treated brain endothelial cells suggesting a clear role of Lcn2 in HMGB1 release though other mediators may not be ruled out. Absence of a systemic knock out of Lcn2 or a liver specific knockout (KO) of Lcn2 limited our interpretation in clearly stating that Lcn2 may be the only mediator affecting HMGB1 secretion in the brain tissues. There are very few studies till date showing induction of HMGB1 by Lcn2. In a recent study, Song et al. reported induction of HMGB1 and activation of NLRP3 inflammasome complex in presence of increased Lcn2 in heart tissue [58]. Notably, our current finding not only shows significant induction of HMGB1 in presence of Lcn2, but it describes Lcn2 as putative molecule acting through liver-brain axis in NASH pathology.

Advancing the mechanism of Lcn2-mediated HMGB1 secretion and release, we further studied the HMGB1-mediated inflammatory pathways in our NASH model. Previous studies have shown that HMGB1induces neuroinflammation by interacting with TLR4 and/or RAGE receptors [59, 60]. Interestingly, we found that there was an increased expression of both the receptors in NASH mice. Binding of HMGB1 to its putative receptors induce severe oxidative stress and cell death [61]. Previously, our own laboratory has shown that HMGB1 induces oxidative stress by interacting with RAGE receptor in intestinal epithelial cells and promotes ectopic intestinal inflammation in MAFLD [33]. In this study, we showed an increased oxidative stress in brain of NASH following HMGB1 induction by Lcn2 (Fig. 4). Therefore, it can be justifiably predicted that Lcn2 might be a key inducer of oxidative stress following HMGB1 secretion in brain under NASH physiology via its interaction with either TLR4/RAGE pathway and that might involve NOX-2, a key generator of highly reactive superoxide radicals and hydrogen peroxide. Oxidative stress induces multiple pro inflammatory signaling pathways in NASH that have been implicated by researchers including our laboratory previously. Activation of p38 mitogen-activated protein kinase (MAPK) is one of the major intracellular signal transducing factors that is activated during oxidative stress [62]. Activation of p38 MAPK is also reported to be involved in AD [63]. Our results showed a significantly increased p38 protein expression in cerebral cortex of MCD fed NASH mouse. Notably, activated p38 MAPK contributes to activation of nuclear factor kappa B (NF-κB) which indeed is most often responsible for activation of NLRP3 inflammasome, cytokine, and chemokines secretion [64]. The NLRP3 inflammasomes are multimeric protein complexes composed of cytosolic sensor NLRP3, bridge protein apoptosis-associated speck-like protein, and cysteine protease caspase1 and have been found among all cell types of the brain. Current results reported here showed activation of NLRP3 in NASH mouse model with increased genomic expression of caspase1 and IL-18 [65]. In addition, abundant evidence exist that suggest that NLRP3 inflammasome is actively involved in the onset and development of neuroinflammatory diseas e[66, 67]. Therefore, our results of an increased IL-6 and IL-1β expression in NASH mice brain tissue is further indicative of an activated NLRP3 inflammasome activation. Furthermore, brain microvascular endothelial cells showed the highest immunoreactivity to NLRP3 inflammasome and might be a strong indicator of inflammatory pathology in the blood-brain barrier in our study. It has been shown that tight junction proteins, especially Claudin 5 in microvascular endothelial cells, maintain BBB integrity [68]. Disruption of Claudin 5 causes leaky BBB that leads to inflammation and dysfunction of the barrier crucial to the maintenance of brain functions. Interestingly, Claudin 5 KO mouse showed increased permeability of BBB to molecules < 800 Da and that caused BBB dysfunction [69]. Our current results implicated a possible evidence of BBB dysfunction by showing the significantly decreased expression of Claudin 5 protein in presence of high circulatory Lcn2 in NASH physiology though more thorough evidence using tight junction leaching would have been appropriate for this study (Fig. 6). Furthermore, results showed a significant increase in the secretion of pro-inflammatory cytokines IL-6 and IL-1β in presence of high circulatory Lcn2 from brain endothelial cells, thus suggesting a strong proinflammatory microenvironment in the BBB.

Role of Lcn2 in BBB dysfunction is somewhat unclear. Increased Lcn2 in brain has been shown to serve as a potent protective factor in the CNS in response to systemic inflammation [70]. However, in a different study, Egaschira reported that Lcn2 caused subarachnoid hemorrhage-induced blood-brain barrier disruption [71]. A recent study also showed a non-disruptive blood-brain barrier dysfunction by Lcn2 following ischemic stroke as shown in human endothelial cells [72]. In agreement of the previous studies showing a strong proinflammatory role of Lcn2 that also was implicated in BBB dysfunction, our study showed that Lcn2 might play a pivotal role in BBB disruption. Additional evidence supporting the above conclusion came from the observation that Claudin 5 protein expression was significantly restored, and a subsequent decrease in proinflammatory cytokines IL-6 and IL-1β secretion was observed in 24p3RsiRNA (a blocked Lcn2 receptor) exposed brain endothelial cells that were co-incubated with Lcn2 or serum of NASH mice. Furthermore, decreased secretion of HMGB1 in 24p3R siRNA exposed brain endothelial cells strongly implicated the role of Lcn2 in HMGB1 secretion and induction of neuroinflammation in the blood-brain barrier interface in NASH physiology.

It is important to note that adipokine leptin induces inflammatory responses in blood brain-barrier cells and in MAFLD and NASH pathology [73]. Knocking down of leptin receptor maintained tight junction integrity and prevented leukocyte extravasation in the spinal cord [74]. Our present study showed Lcn2 as a more potent proinflammatory mediator in neuronal tissues of NASH mice when compared to leptin. This observation is stemmed from the fact that Lcn2 was able to cause a significantly increased expression of inflammatory cytokines TNFα, IL-1β, and matrix metalloprotease 9 (MMP9) when compared to leptin (Supplementary Fig.1).

## Conclusions

Taken together, our present study identifies Lcn2 as a putative molecule for neuroinflammation and neurodegeneration in MAFLD by acting through liver-brain axis. Further, high circulatory Lcn2 in MAFLD induced NLRP3 inflammasome activation following induction of HMGB1 secretion in the brain and caused blood-brain barrier dysfunction by the way of altered expression of tight junction protein Claudin5 and secretion of pro-inflammatory cytokines IL-6 and IL-1β from brain endothelial cells. The higher Lcn2 was also implicated in decreased BDNF and a subsequent increase in Tau protein respectively suggesting an overall neurotoxic pathology. Our studies reported here may help design new therapeutic approaches to counter the neuroinflammatory pathology in NASH but also in other related brain pathology associated with chronic inflammatory diseases.

## Supplementary information

**Additional file 1: Supplementary Fig. 1.** A. Real time PCR expression of TNFα, IL-1β, MMP 9, VCAM1, ICAM 1 with brain endothelial cells exposed with Vehicle (CONTROL), Mouse recombinant Lcn2, Mouse recombinant leptin, and with Lcn2 and leptin both. Data was normalized with 18S rRNA expression and fold change was calculated with respect to CONTROL. Significance was calculated by one way ANOVA ( *** p < 0.001). B. Bar graph of normalized expression of ICAM1 and VCAM1, represented as ratio (ICAM1/VCAM1) of A. Data was represented as mean ± SD (n = 3), Significance was calculated by unpaired t-test with respect to CONTROL, * p < 0.05, ** p < 0.01, *** p < 0.001. **Supplementary Fig. 2.** A. Claudin 5 immunoreactivity displayed by immunoblot in mouse primary brain endothelial cells and 24p3RsiRNA exposed brain endothelial cells followed by treating with mouse recombinant Lcn2 and serum from MCD fed mouse group, and with vehicle (0.05% DMSO). B. Densitometric quantification of western blot in D, data was normalized with β actin, and expressed as mean ± SD, significance was calculated by paired t test between the groups, * p < 0.05, ** p < 0.01, *** p < 0.001.

## Data Availability

The datasets used and/or analyzed during the current study are available from the corresponding author on reasonable request.
