# Supplementary material for: Lipocalin 2 induces neuroinflammation and blood-brain barrier dysfunction through liver-brain axis in murine model of nonalcoholic steatohepatitis
Source: J Neuroinflammation. 2020 Jul 4;17:201. doi: 10.1186/s12974-020-01876-4 (PMC7335438; doi:10.1186/s12974-020-01876-4)
Supplement: Supplementary file 1 — Additional file 1: Supplementary Fig. 1. A. Real time PCR expression of TNFα, IL-1β, MMP 9, VCAM1, ICAM 1 with brain endothelial cells exposed with Vehicle (CONTROL), Mouse recombinant Lcn2, Mouse recombinant leptin, and with Lcn2 and leptin both. Data was normalized with 18S rRNA expression and fold change was calculated with respect to CONTROL. Significance was calculated by one way ANOVA ( *** p < 0.001). B. Bar graph of normalized expression of ICAM1 and VCAM1, represented as ratio (ICAM1/VCAM1) of A. Data was represented as mean ± SD (n = 3), Significance was calculated by unpaired t-test with respect to CONTROL, * p < 0.05, ** p < 0.01, *** p < 0.001. Supplementary Fig. 2. A. Claudin 5 immunoreactivity displayed by immunoblot in mouse primary brain endothelial cells and 24p3RsiRNA exposed brain endothelial cells followed by treating with mouse recombinant Lcn2 and serum from MCD fed mouse group, and with vehicle (0.05% DMSO). B. Densitometric quantification of western blot in D, data was normalized with β actin, and expressed as mean ± SD, significance was calculated by paired t test between the groups, * p < 0.05, ** p < 0.01, *** p < 0.001. [file 12974_2020_1876_MOESM1_ESM.pptx]

## Slide 1
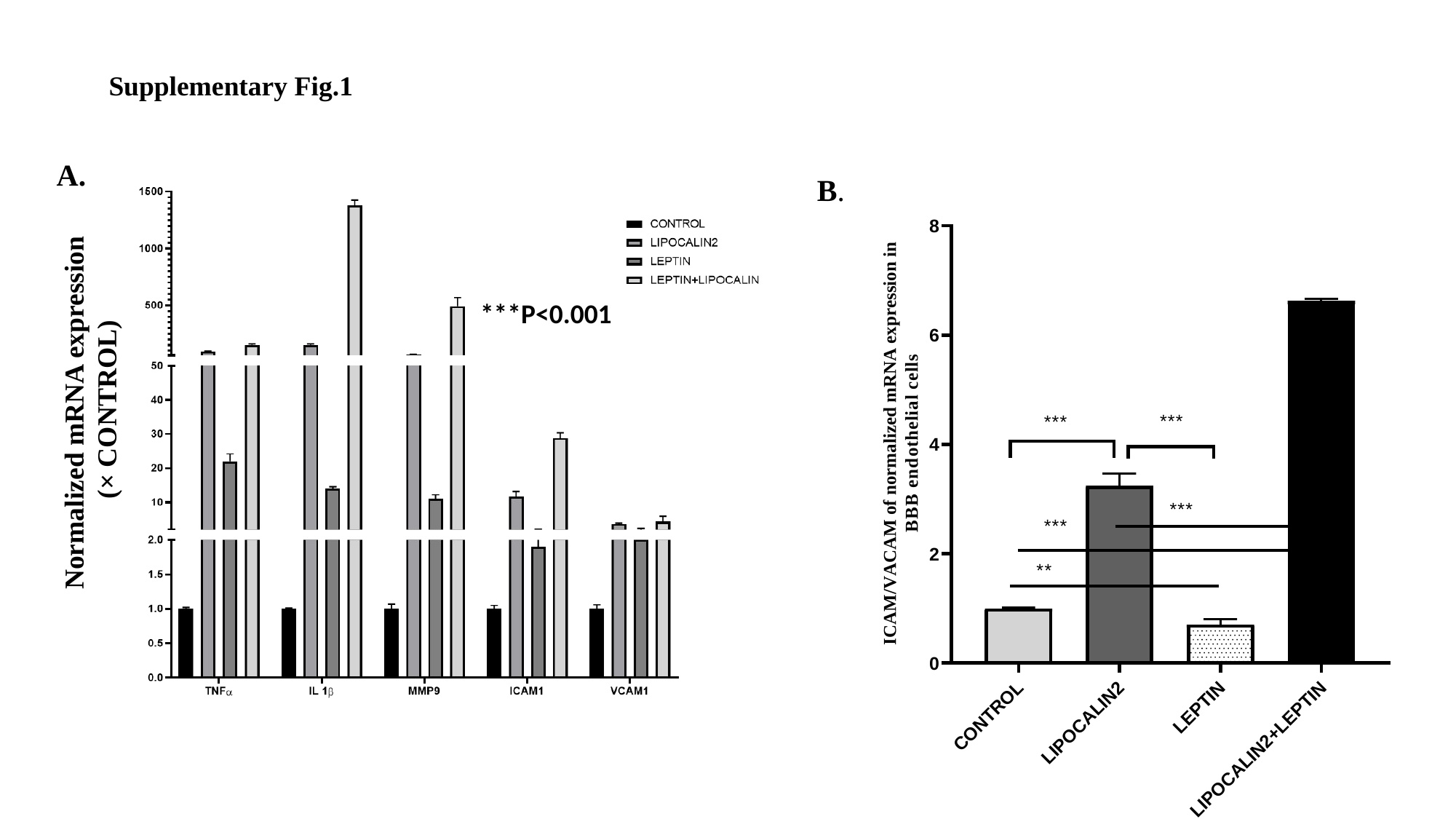

Supplementary Fig.1
A.
B.
***P<0.001
Normalized mRNA expression
(× CONTROL)

## Slide 2
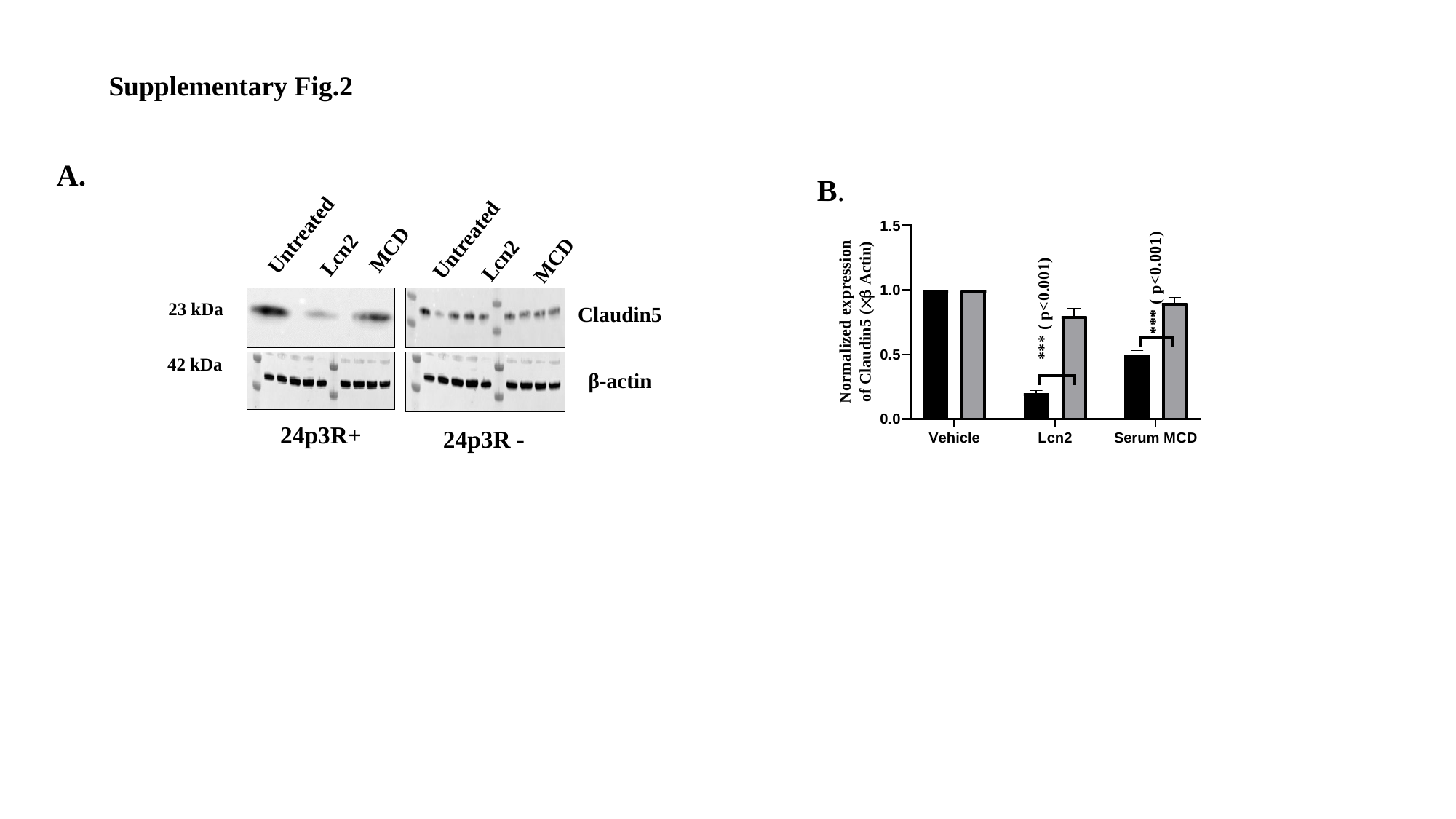

Supplementary Fig.2
A.
B.
Lcn2
Untreated
MCD
Lcn2
MCD
23 kDa
Claudin5
42 kDa
β-actin
24p3R+
24p3R -
Untreated
